# Geographic range is a poor predictor of high-temperature responses among conifers in the boreal–temperate ecotone varying in shade tolerance

**DOI:** 10.1093/treephys/tpag014

**Published:** 2026-02-21

**Authors:** William R Vaughn, Anthony R Taylor, David A MacLean, Loïc D’Orangeville, Chris B Edge, Robert W Buchkowski

**Affiliations:** Natural Resources Canada, Canadian Forest Service - Atlantic Forestry Centre, 1350 Regent Street, PO Box 4000, Fredericton, NB E3B 5P7, Canada; Faculty of Forestry and Environmental Management, University of New Brunswick, 28 Dineen Drive, Fredericton, NB E3B 5A3, Canada; Faculty of Forestry and Environmental Management, University of New Brunswick, 28 Dineen Drive, Fredericton, NB E3B 5A3, Canada; Faculty of Forestry and Environmental Management, University of New Brunswick, 28 Dineen Drive, Fredericton, NB E3B 5A3, Canada; Department of Wood and Forest Sciences, Laval University, 2405 rue de la Terrasse, Québec, QC G1V 0A6, Canada; Natural Resources Canada, Canadian Forest Service - Atlantic Forestry Centre, 1350 Regent Street, PO Box 4000, Fredericton, NB E3B 5P7, Canada; Faculty of Forestry and Environmental Management, University of New Brunswick, 28 Dineen Drive, Fredericton, NB E3B 5A3, Canada; Department of Biology, Western University, 1151 Richmond Street, London, ON N6A 3K7, Canada

**Keywords:** boreal trees, climate warming, heatwave, photosynthesis, temperate trees, thermotolerance

## Abstract

Projected warming and heatwave frequency may disproportionately impact growth and survival of northern tree species at their southern range limits in the boreal–temperate ecotone in North America. However, the extent to which geographic range and shade tolerance influence species’ responses to warming remains uncertain. We investigated the effects of 12 levels of ex situ warming on growth, mortality and physiology of seven tree species from the boreal–temperate ecotone, each with different southern range limits and shade tolerances. White pine (*Pinus strobus* L.), a southern temperate conifer, maintained photosynthetic capacity following prolonged warming and displayed the highest peak growth temperature. However, despite sharing a similar southern range limit, shade-tolerant eastern hemlock (*Tsuga canadensis* (L.) Carrière) exhibited negligible growth responses and significant foliar damage under warming. Jack pine (*Pinus banksiana* Lamb.), a light-demanding northern boreal conifer, showed much less foliar damage but reduced photosynthetic capacity under warming. Shade-tolerant species exhibited greater foliar damage and mortality under high temperatures, while light-demanding species exhibited more tolerance regardless of range limits. We conclude that while geographic range explains some responses to warming, shade tolerance may be equally important. These findings provide empirical data to improve forest model accuracy and inform management strategies for forest regeneration and ecosystem stability.

## Introduction

Under the intermediate- and high-emission scenarios (Shared Socio-economic Pathways 2-4.5 and 5-8.5), mean global surface temperatures are projected to rise by 2.1–3.5 °C and 3.3–5.7 °C, respectively, by the end of this century (IPCC 2023). This warming is expected to occur at a much faster rate in higher-latitude ecosystems (Zhang et al. 2019), like the boreal–temperate ecotone found throughout most of the northern hemisphere (Pastor and Mladenoff 1992, Goldblum and Rigg 2010). The direction and magnitude of the responses to future warming in boreal and temperate tree species co-occurring in this transitional zone have been linked to their geographic ranges (Reich et al. 2015, Evans and Brown 2017, Montgomery et al. 2020, Reich et al. 2022). Specifically, warming may hinder regeneration, growth, net photosynthetic carbon gains and survival in boreal species growing near the southern edge of their range while enhancing these processes in temperate species growing near their northern range limits (Fisichelli et al. 2014*a*, 2014*b*, Reich et al. 2015, Wright et al. 2018, Reich et al. 2022).

Future warming will impact carbon assimilation through its effects on photosynthesis. Under short-term climate warming projections, some species may experience greater carbon gains (Taylor et al. 2017, D’Orangeville et al. 2018, Vaughn et al. 2021, Reich et al. 2022). As temperatures rise within the optimum temperature range of photosynthesis, the rates of both photosynthesis and respiration are initially enhanced due to increases in enzymatic activity (Way and Oren 2010). Over longer periods of warming, however, photosynthesis exhibits less thermal acclimation than respiration, leading to an increase in net carbon gain, as carbon loss through respiration is reduced while carbon gain through photosynthesis remains relatively elevated (Campbell et al. 2007, Way and Sage 2008*a*, 2008*b*, Crous et al. 2022). When temperatures rise beyond the photosynthetic temperature optimum, which is typically between 20 and 30 °C for temperate and boreal species (Dreyer et al. 2001, Sage et al. 2008, Sendall et al. 2015), net carbon assimilation begins to decline, resulting from decreasing stomatal conductance, Rubisco activity and photosystem II (PSII) efficiency (Haldimann and Feller 2004, Perdomo et al. 2017, Dusenge et al. 2021), and increasing respiration, photorespiration and photoinhibition (Mathur et al. 2014, Teskey et al. 2015, Guha et al. 2018).

Temperatures above 40 °C, such as those that occur during increasingly frequent heatwaves (Teskey et al. 2015, Lu et al. 2023, Yin et al. 2023), can cause irreversible damage to PSII reaction centers (Yordanov 1992, Kalaji et al. 2017, Petrik et al. 2023) and foliage (Colombo and Timmer 1992). While the growth and physiology of boreal tree species have been investigated under moderate warming (<40 °C; Fisichelli et al. 2014*a*, 2014*b*, Carles et al. 2015, Reich et al. 2015, Wright et al. 2018, Vaughn et al. 2021, Reich et al. 2022), the impacts of heatwave conditions (>40 °C) have received less attention. For example, the Spruce and Peatland Responses Under Changing Environments experiment (Hanson et al. 2017) examined only black spruce (*Picea mariana* (Mill.) B.S.P) and tamarack (*Larix laricina* (Du Roi) K. Koch) under four warming treatments, with maximum temperatures reaching 41 °C. Notably, this experiment observed a divergence in the stomatal response to warming between these two species and no significant interaction between warming and CO_2_ for any measured response (Dusenge et al. 2021, 2024).

Although warming generally enhances photosynthetic rates in trees growing under suboptimal temperatures, the photosynthetic acclimation potential and the threshold for heat-induced stress differ among species and plant functional types (Reich et al. 2015, Teskey et al. 2015, Guha et al. 2018, Dusenge et al. 2019, Vaughn et al. 2021, Crous et al. 2022). Moreover, the degree to which stomatal closure limits photosynthesis has been linked to a species’ sensitivity to soil moisture and temperature (Dusenge et al. 2021, Reich et al. 2022). Intraspecific variations in these characteristics may exist within species throughout their range (Hamrick et al. 1992). However, in the context of climate warming in the boreal–temperate ecotone, adaptation to warming in leading-edge (northern) populations of temperate species may be facilitated by gene flow from central populations in warmer areas, whereas trailing-edge (southern) populations of boreal species are experiencing climatic conditions not encountered anywhere within their current range (Hu and He 2006). Thus, the flow of warm-adapted genes from central populations of boreal species is not possible, which limits the capacity to respond positively to increasing temperatures (Kremer et al. 2012). This lack of gene flow may explain why regeneration and growth of northern boreal tree species are expected to decline under future warming in the boreal–temperate ecotone relative to more southern, warm-adapted temperate species (Fisichelli et al. 2014*a*, 2014*b*, Reich et al. 2015, 2022, Evans and Brown 2017, Wright et al. 2018).

Under adequate soil moisture, stomatal response to extreme heat events remains unresolved (Urban et al. 2017). Past studies have reported conflicting results, possibly due to interactions among vapor pressure deficit, soil water availability and the degree of isohydricity (Weston and Bauerle 2007, Cerasoli et al. 2014, Urban et al. 2017). For example, under adequate soil water availability, white pine (*Pinus strobus* L.) maintained stomatal conductance under 51 °C and a vapor pressure deficit of 7.3 kPa (Guha et al. 2018), while red maple (*Acer rubrum* L.) exhibited a steady decline in stomatal conductance with rising temperature above 30 °C (Weston and Bauerle 2007). Maintaining transpiration during extreme heat events reduces foliar temperature through evaporative cooling, thereby minimizing thermal stress (Kolb and Robberecht 1996, Birami et al. 2018), while risking xylem embolism and hydraulic failure (McDowell et al. 2008). However, very little research has been done regarding the influence of high temperature on the transpiration of coniferous species in the boreal forest.

Tree species varying in shade tolerance may respond differently to rising temperatures. Under similar light conditions, shade-tolerant tree species contain higher foliar concentrations of potentially toxic reactive oxygen species (ROS) due to their lower energy-utilizing capacity relative to light-demanding species (Hansen et al. 2002). An imbalance between energy capture and utilization results in the production of ROS, as excess electrons from the photosynthetic electron transport chain begin to reduce oxygen molecules (Asada 1999), a process that is intensified under high temperatures (Sairam et al. 2000, Larkindale and Knight 2002). Light-demanding species, like jack pine (*Pinus banksiana* Lamb.), mitigate oxidative damage better than shade-tolerant species due to greater photochemical energy utilization (Hansen et al. 2002) and enhanced mechanisms that protect cells from the toxic effects of ROS (Cai et al. 2005, Favaretto et al. 2011). In the boreal–temperate ecotone, oxidative damage sustained during heatwave events may therefore be more prominent in shade-tolerant species, like balsam fir (*Abies balsamea* (L.) Mill.), eastern hemlock (*Tsuga canadensis* (L.) Carrière) and red spruce (*Picea rubens* Sarg.).

In this paper, we used phytotrons to investigate the effects of ex situ warming over 2 years on seedlings of seven coniferous tree species with varying southern range limits and shade tolerances: balsam fir, black spruce, eastern hemlock, jack pine, red spruce, white pine and white spruce (*Picea glauca* (Moench) Voss). We hypothesized that: (i) southern species—eastern hemlock and white pine—would exhibit optimal growth under higher temperatures than northern species, as they are adapted to warmer climates; (ii) greater transpiration rates under warming would limit heat-induced foliar damage through evaporative cooling; and (iii) shade-tolerant species would experience greater heat stress than light-demanding species, as their higher susceptibility to oxidative damage under similar light conditions is exacerbated by rising temperatures.

## Materials and methods

### Study species

The study species included five that are predicted to decline in abundance by the end of the 21^st^ century under Shared Socio-economic Pathway 5-8.5—balsam fir, black spruce, jack pine, red spruce and white spruce; a species that is predicted to experience minimal effects—eastern hemlock; and a species that is expected to increase in abundance—white pine (Rogers et al. 2017, Taylor et al. 2017). These tree species coexist in North America’s boreal–temperate ecotone, which stretches from Minnesota and Wisconsin in the west, through the Great Lakes, Central Ontario, southern Québec and into the Maritime provinces (Goldblum and Rigg 2010). However, their distributions differ geographically, with the ranges of eastern hemlock and white pine extending much farther south than those of balsam fir, black spruce, jack pine, red spruce and white spruce (Table 1). Additionally, shade tolerance varies among the chosen species with jack pine showing the least tolerance (1.36; 0 (no tolerance) to 5 (maximal tolerance)) and balsam fir exhibiting the most tolerance (5.01; Niinemets and Valladares 2006).

**Table 1:** Core range limit latitudes and corresponding mean annual temperatures (MATs) for all seven study species ordered by ascending southern core range limit latitude (McKenney et al. 2007). For each species, MAT values for the northern and southern range limits are equivalent to the 2.5^th^ and 97.5^th^ percentiles of the occupied MAT range, respectively. Shade tolerance index (Niinemets and Valladares 2006): 0 (no tolerance) to 5 (maximal tolerance). The shade tolerance index was included to support the interpretation of the results presented in the discussion.

Common name	Southern core range limit		Northern core range limit		Shade tolerance
	Latitude	MAT (°C)	Latitude	MAT (°C)	
Eastern hemlock	36.36797	12.3	47.94462	2.7	4.83
White pine	36.37930	12.8	51.11618	1.2	3.21
Red spruce	41.78465	7.4	49.35338	1.3	4.39
Balsam fir	41.98786	6.7	62.89110	−2.2	5.01
White spruce	43.78611	6.5	64.12388	−4.8	4.15
Black spruce	43.88313	6.1	63.84247	−4.0	4.08
Jack pine	44.39451	6.5	63.74842	−3.1	1.36

### Seed source and seedling preparation

All seedlings were grown from seed harvested in New Brunswick, sourced either from a single-tree collection (eastern hemlock—National Tree Seed Centre, Fredericton, New Brunswick, Canada), bulk seed collections from wild stands (balsam fir, black spruce and white pine—National Tree Seed Centre; jack pine—Kingsclear Nursery, Island View, New Brunswick, Canada; red spruce—J.D. Irving Ltd, Juniper, New Brunswick, Canada) or a bulk seed collection from a tree improvement program (white spruce—J.D. Irving Ltd). Seedlings were grown for a full year prior to the experiment and then re-potted during April of 2022 in 3.8 L pots with a mixture of peat moss, perlite, loam and aggregate, which allowed adequate drainage to be maintained over the 2-year experiment.

During each growing season, seedlings were fertilized with water-soluble 11:41:08 (N:P:K) starter fertilizer (Plant Products Inc., Leamington, Ontario, Canada) at a concentration of 50 p.p.m. N once every 2 weeks prior to bud flush. Once buds flushed, seedlings were fertilized with water-soluble 20:08:20 (N:P:K) grower fertilizer (Plant Products Inc., Leamington, Ontario, Canada) at a concentration of 100 p.p.m. N every 2 weeks until late August. After this time, a water-soluble finisher fertilizer (08:20:30 (N:P:K); 35 p.p.m. N; Plant Products Inc., Leamington, Ontario, Canada) was used every 2 weeks until the end of the growing season.

### Experimental design

Seedling growth, mortality and physiological impacts were measured under 12 different temperature treatments applied within 12 temperature-controlled phytotrons (Figure S1.1 available as Supplementary Data at *Tree Physiology* Online) over two growing seasons. At the beginning of the experiment, 10 1-year-old seedlings of each species, totaling 70 seedlings, were placed in single-species groups (to minimize light competition among species) in each of the 12 phytotrons. The species groups were rotated within the same phytotron weekly to account for microenvironmental variation within each phytotron. Seedlings were watered when soil moisture was ≤15% volumetric water content to eliminate the negative effects of low soil water availability (Vaughn et al. 2021). Light levels in each phytotron were ~600 μmol m^−2^ s^−1^ Photosynthetic photon flux density irradiance at plant height. The same seedlings were used throughout the entire experiment with no replacements.

The temperature schedule for the coldest treatment (control) was produced by calculating mean hourly temperature for each day of the growing season using 30 years (1991–2020) of temperature data collected from the Fredericton International Airport weather station located in Lincoln, New Brunswick, Canada at 45.87° N and 66.54° W. Temperature data from this location were used to create the control treatment, as it is approximately centered, latitudinally, within the boreal–temperate ecotone. These daily temperature data were then aggregated by week to produce 24-h temperature schedules that were repeated for each day of the corresponding week in the growing season. The warmest treatment (treatment 12) was consistently 18 °C above the control. The 18 °C temperature difference was chosen to ensure that seedlings in the warmest treatment would experience temperatures that approach the critical threshold of PSII thermostability during the hottest period of the growing season (Schreiber and Berry 1977, Kalaji et al. 2017, Petrik et al. 2023). Ten other treatments were evenly spaced at ~1.6 °C increments between the control and treatment 12 (see Table 2 for further treatment details). During the growing seasons, the phytotron temperature controllers were reprogrammed every week with new 24-hour temperature schedules, mimicking diurnal and seasonal temperature patterns.

**Table 2:** Description of the 12 treatments showing the average summer treatment temperature (mean temperature from 20 June to 22 September), degrees above control, maximum experienced temperature of each treatment and growing season period descriptions.

Treatment	Average summer temperature (°C)	Degrees above control (°C)	Maximum treatment temperature (°C)	Growing season period group	2022 Growing season^1^		2023 Growing season^1^	
					Start	End	Start	End
1 (control)	18.2	0.0	25.0	1	05–12	09–12	05–22	09–18
2	19.8	1.6	26.6	1	05–12	09–12	05–22	09–18
3	21.5	3.3	28.3	1	05–12	09–12	05–22	09–18
4	23.1	4.9	29.9	2	05–12	09–26	05–01	10–02
5	24.7	6.5	31.5	2	05–12	09–26	05–01	10–02
6	26.4	8.2	33.2	2	05–12	09–26	05–01	10–02
7	28.0	9.8	34.8	3	05–12	10–10	04–10	10–16
8	29.7	11.5	36.4	3	05–12	10–10	04–10	10–16
9	31.3	13.1	38.1	3	05–12	10–10	04–10	10–16
10	32.9	14.7	39.7	4	05–12	10–24	03–20	10–30
11	34.6	16.4	41.3	4	05–12	10–24	03–20	10–30
12	36.2	18.0	43.0	4	05–12	10–24	03–20	10–30

^1^Month–day format.

Schweiger et al. (2016) observed that 10–15 predictor levels along the gradient being studied (e.g., temperature), combined with three observations per predictor level, maximized prediction success for scenarios with moderate to high levels of random variation. These gradient designs are becoming more popular, as classical replicated designs often fail to capture non-linearities of ecological responses (Kreyling et al. 2018). In our study, rather than replicating fewer temperature treatments in multiple phytotrons, we chose to maximize sampling frequency along the temperature gradient (12 temperature levels, each assigned to 1 of 12 phytotrons), which allowed us to observe biological responses to temperature change with greater resolution. Moreover, by applying temperatures beyond those projected in climate change scenarios, we were able to clearly define temperature-induced foliar damage and mortality thresholds of all seven tested species.

In the first year of the experiment, all temperature treatments began on 12 May 2022, as seedlings were not prepared until this time. The 12 treatments were divided into four growing season periods of varying durations (Table 2), with warmer treatments having longer growing seasons to mimic projected climate-driven extensions (Zhang et al. 2019). End dates for the 2022 growing season were offset by 2 weeks: the first growing season period, containing the three coolest treatments, ended on 12 September, whereas the fourth period, containing the three warmest treatments, ended on 24 October. These dates were selected to ensure that seedlings in the warmest treatments would receive satisfactory chilling time over the winter. To ensure all seedlings were frost-hardy prior to being stored outdoors after the end of the first growing season, seedlings were placed in a walk-in fridge (3 °C) from 4 p.m. to 8 a.m. for 10 days to expose the seedlings to a reduced photoperiod and cold temperatures, which have been shown to stimulate frost hardening (Mellerowicz et al. 1992). Treatment start dates in the 2023 growing season were separated by 3 weeks, starting on 20 March for the fourth growing season period (warmest treatments), mimicking an earlier growing season (Zhang et al. 2019), and 22 May for the first (coolest treatments); end dates were separated by 2 weeks, starting on 18 September.

### Seedling measurements, damage and mortality

Seedling height and diameter (measured at the root collar) were recorded at the beginning and end of the first growing season and at the end of the second growing season to determine yearly growth increment. Four seedlings of each species/treatment were randomly selected for above-ground biomass measurements at the ends of each growing season. Biomass samples were oven-dried at 70 °C for 48 h (Taylor et al. 2007) before being weighed. Seedling growth and biomass measurements were used to determine optimal growth temperatures for each species. To assess species’ thermal sensitivities, foliar damage scores (visual evaluation of needle vigor; 0: no damage; 1: <⅓ damage; 2: ≥⅓ and <⅔ damage; 3: ≥⅔ damage; 4—dead; see Ravn et al. 2022) of current year needles and seedling mortality were recorded at the ends of both growing seasons. Seedlings were considered dead if they lacked both live foliage and green vascular cambium tissue. Dead seedlings were removed from the experiment and not replaced.

### Gas exchange

Using a LI-6800 portable photosynthesis system with a large leaf and needle chamber (LI-COR, Lincoln, NE, USA), thermal response measurements of net photosynthesis (*A_net_*), stomatal conductance (*g_s_*) and transpiration (*E*) were collected from fully expanded, current-year foliage during the second growing season. These data were used to determine the impacts of warming on carbon assimilation among species and to compare transpiration among species to assess the ameliorative effects of high transpiration rates on heat-induced foliar damage. Three seedlings each of black spruce, jack pine, white pine and white spruce were randomly selected from four treatments evenly spaced across the treatment temperature range (1, 4, 7 and 10; Table 2). These four species exhibited the least damage under the warmest treatments after the first growing season and winter, so measuring their gas exchange would provide physiological insight into their comparatively greater ability to withstand thermal stress. The randomly selected seedlings were measured during the warmest week of the second year of the experiment (24–30 July 2023). Each seedling was placed inside a small temperature-controlled chamber along with the LI-6800 machine. The temperature in the small chamber was set to match the LI-6800 cuvette temperature to ensure that the whole seedling experienced the same degree of warming. For each seedling, gas exchange measurements were performed in the LI-6800 cuvette at four sample temperatures (24.9, 29.8, 34.7 and 39.6 °C) under saturating photosynthetically active radiation (1000 μmol m^−2^ s^−1^), 60% relative humidity and 400 μmol mol^−1^ CO_2_. Each seedling remained in the cuvette, while the temperature was increased sequentially from the coldest to the warmest sample temperature. Seedling *A_net_* and *g_s_* were allowed to stabilize at each sample temperature prior to logging. After gas exchange measurements, projected leaf area was determined by processing scanned images of every sample’s foliage with ImageJ (Schneider et al. 2012).

### Statistical analyses

Species-specific regression analyses were conducted to measure the effect of temperature treatment on seedling height and diameter increment and above-ground biomass for each growing season. While data from both growing seasons were analyzed, the results focus on data collected after the second growing season. Growth during the first growing season may have been influenced by predetermined growth that developed prior to the experiment, which has been shown to mask the effect of warming (Ravn et al. 2023). Models were fit using the *poly* function from the *stats* package in *R* (R Core Team 2024) to determine the effect of both the linear and quadratic terms of the explanatory variable, temperature. The *poly* function creates orthogonal polynomial terms and avoids the collinearity issues associated with raw polynomials. Additionally, to evaluate whether the more complex model provided a significantly better fit to the data, models were compared using an *F*-statistic and the corrected Akaike Information Criterion (AICc, *AICc* function from the *MuMIn* package (Bartoń 2024)). Model fit was further evaluated by calculating *R*^2^ (unadjusted), which measures the proportion of variance in the response variable explained by the model. Residual normality and homoscedasticity of the models were evaluated with the Shapiro–Wilk test and the Breusch–Pagan test, respectively.

Species’ damage scores for each growing season were analysed with ordinal logistic regression models using the *clm* function from the *ordinal* package (Christensen 2023). Model fit was evaluated by calculating McFadden’s pseudo-*R*^2^ for each growing season model. The probability of each damage score was determined for every species in every temperature treatment using the *ggpredict* function from the *ggeffects* package (Lüdecke 2018).

Seedling mortality was analyzed with a two-parameter log-logistic model using the *drm* function from the *drc* package (Ritz and Strebig 2022), and the lethal dose of temperature that caused 50% mortality (LD50) was calculated for each species. Weight was set to the total number of seedlings (less the amount removed for biomass measurements) per species per treatment, type was set to binomial distribution and upper and lower limits were set to 1 and 0. To determine whether the addition of species as a grouping factor was warranted, we compared the full model that included species with the null model using a likelihood ratio test. Significant differences among species’ LD50s were determined using the *EDcomp* function from the *drc* package.

To assess whether species’ heat damage thresholds were related to their shade tolerance or geographic distribution, we used linear regression to model the temperature at which foliar damage first occurred for each species as a function of their latitudinal southern range limits and shade tolerances, analyzed in separate models. For each model, we extracted the *P*-value and *R*^2^ to evaluate the strength and significance of each predictor.

Gas exchange results were analyzed with linear mixed-effects models for each response variable using the *lme4* package (Bates et al. 2015). Response variables included *A_net_*, *g_s_* and *E*, while the explanatory variables included treatment temperature (average summer temperature of each treatment, Table 2), sample temperature, and species as fixed effects with seedling ID and phytotron as random effects. Repeated measures of gas exchange were taken from each seedling, which necessitated the inclusion of seedling ID as a random variable. As we only used 4 of the 12 phytotrons, we included the random effect of phytotron in all gas exchange models to account for potential variability among phytotrons that could have influenced the measurements. Marginal and conditional *R*^2^ values were computed for each model to assess model fit. *A_net_* models were species-specific, included linear and quadratic terms of the sample temperature variable, and were evaluated using the *AICc* function to determine the best-fitting model. Post-hoc multiple comparisons among treatment temperatures and sample temperatures were produced for all gas exchange variables and species using the *emmeans* function from the *emmeans* package (Lenth 2021).

## Results

### Species growth response to warming

**Figure 1:**
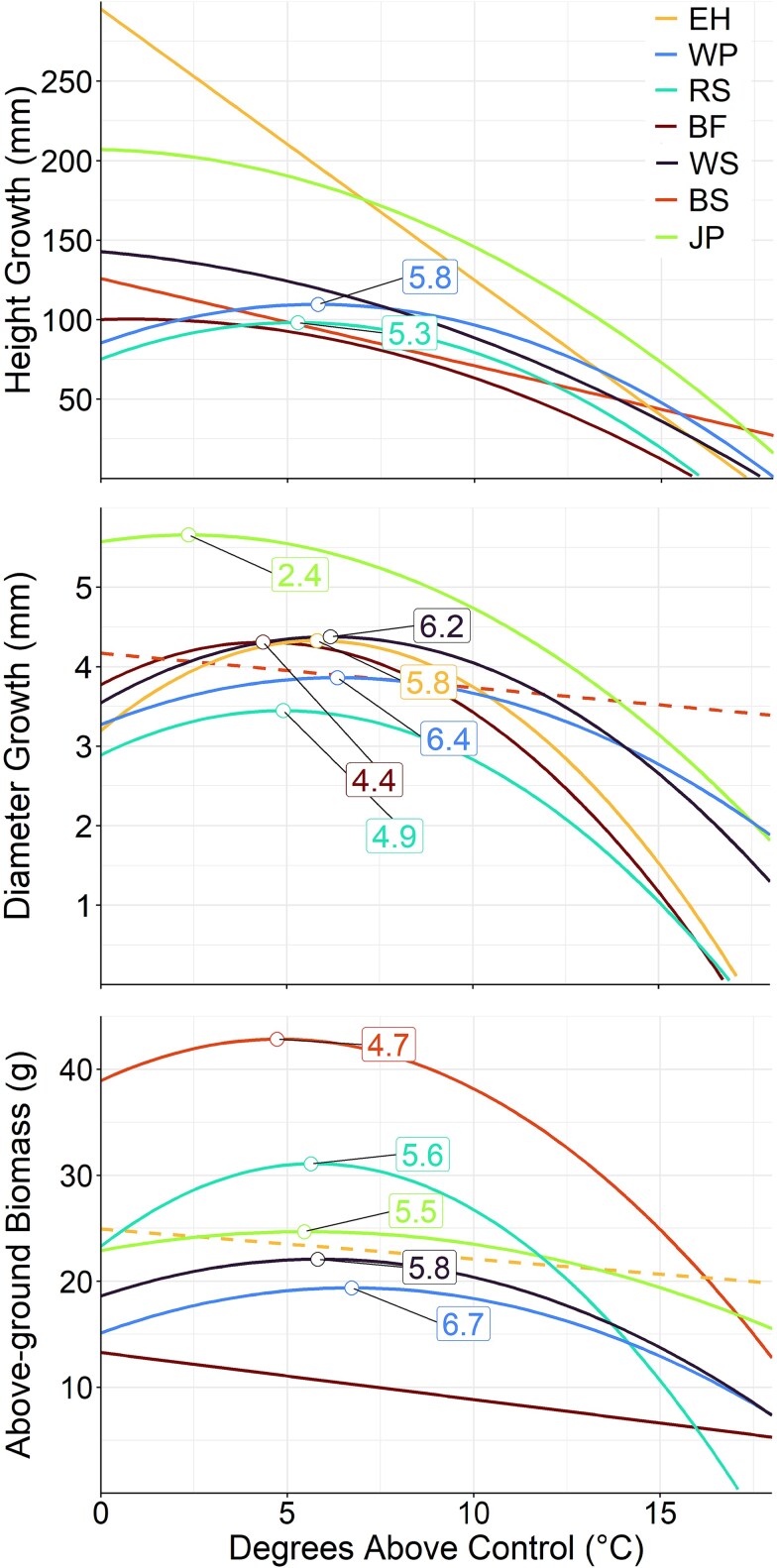
Model-predicted annual height and diameter growth increments, and above-ground biomass of balsam fir (BF), black spruce (BS), eastern hemlock (EH), jack pine (JP), red spruce (RS), white pine (WP) and white spruce (WS) seedlings under 12 levels of warming (control to 18 °C above control) following the second growing season. Solid lines represent significant effects (*P* < 0.05). Numerical labels associated with the response variable curves indicate the temperature above control at which the maximum values occurred (within the displayed area).

Height and diameter models generally performed well, with *R*^2^ values exceeding 0.4, except for height growth in black (*R*^2^ = 0.22) and red (*R*^2^ = 0.21; Table 3) spruce. In contrast, biomass models explained less variation overall, with *R*^2^ values below 0.4 for balsam fir, black spruce, jack pine and white spruce. For all growth response variables, white pine consistently exhibited model-predicted growth maxima at higher temperatures than all other species after the second growing season (Figure 1), demonstrating the greatest potential for improved growth with warming. In contrast, balsam fir and eastern hemlock generally showed either negative or insignificant responses to warming (Figure 1). A potential for improved growth was usually demonstrated by the presence of a significant quadratic term for temperature in a species’ model (Table 3). For height growth, only red spruce and white pine showed positive effects of warming up to 5.3 and 5.8 °C above the control, respectively (Figure 1). However, for diameter growth increment (i.e., secondary growth), jack pine, balsam fir, red spruce, eastern hemlock, white spruce and white pine all showed gains under warming up to 2.4, 4.4, 4.9, 5.8, 6.2 and 6.4 °C above the control, respectively; and, for above-ground biomass, black spruce, jack pine, red spruce, white spruce and white pine exhibited increases up to 4.7, 5.5, 5.6, 5.8 and 6.7 °C above the control, respectively (Figure 1). Descriptions of species-specific growth models for the first growing season can be found in Table S1.1 available as Supplementary Data at *Tree Physiology* Online.

**Table 3:** The 2023 growth variable model comparisons between linear ($T$) and quadratic ($T+{T}^2$) model forms. Models were compared using the corrected Akaike Information Criterion (AICc) to assess model parsimony, and an *F*-statistic was used to evaluate whether the more complex model provided a significantly better fit to the data. Bolded AICc values indicate the best-fitting model, which was further evaluated using *R*^2^. NA indicates no significant effect of temperature treatment.

Species	AICc		*F*-statistic	*P*-value	*R* ^2^
	$\boldsymbol{T}$	$\boldsymbol{T}+{\boldsymbol{T}}^{\mathbf{2}}$			
Height increment					
Eastern hemlock	**730.3**	731.0	1.53	0.221	0.65
White pine	709.0	**692.1**	21.15	**<0.001**	0.43
Red spruce	684.3	**679.8**	6.91	**0.011**	0.21
Balsam fir	579.2	**576.8**	4.60	**0.036**	0.47
White spruce	671.3	**668.6**	4.93	**0.030**	0.63
Black spruce	**669.4**	671.6	0.08	0.780	0.22
Jack pine	726.8	**724.1**	4.92	**0.030**	0.54
Diameter increment					
Eastern hemlock	239.3	219.6	24.91	<0.001	0.44
White pine	162.3	**142.8**	24.47	**<0.001**	0.41
Red spruce	197.7	**185.1**	15.98	**<0.001**	0.42
Balsam fir	193.3	**181.4**	15.21	**<0.001**	0.45
White spruce	218.9	**201.3**	22.03	**<0.001**	0.40
Black spruce	NA	NA	NA	NA	NA
Jack pine	228.6	**224.1**	6.73	**0.012**	0.44
Above-ground biomass					
Eastern hemlock	NA	NA	NA	NA	NA
White pine	269.4	**245.3**	33.24	**<0.001**	0.57
Red spruce	312.3	**300.0**	16.51	**<0.001**	0.48
Balsam fir	**197.1**	199.6	0.03	0.863	0.31
White spruce	296.0	**290.3**	8.23	**0.006**	0.29
Black spruce	329.9	**327.3**	5.00	**0.031**	0.27
Jack pine	275.5	**273.4**	4.51	**0.040**	0.21

### Seedling damage and mortality

**Figure 2:**
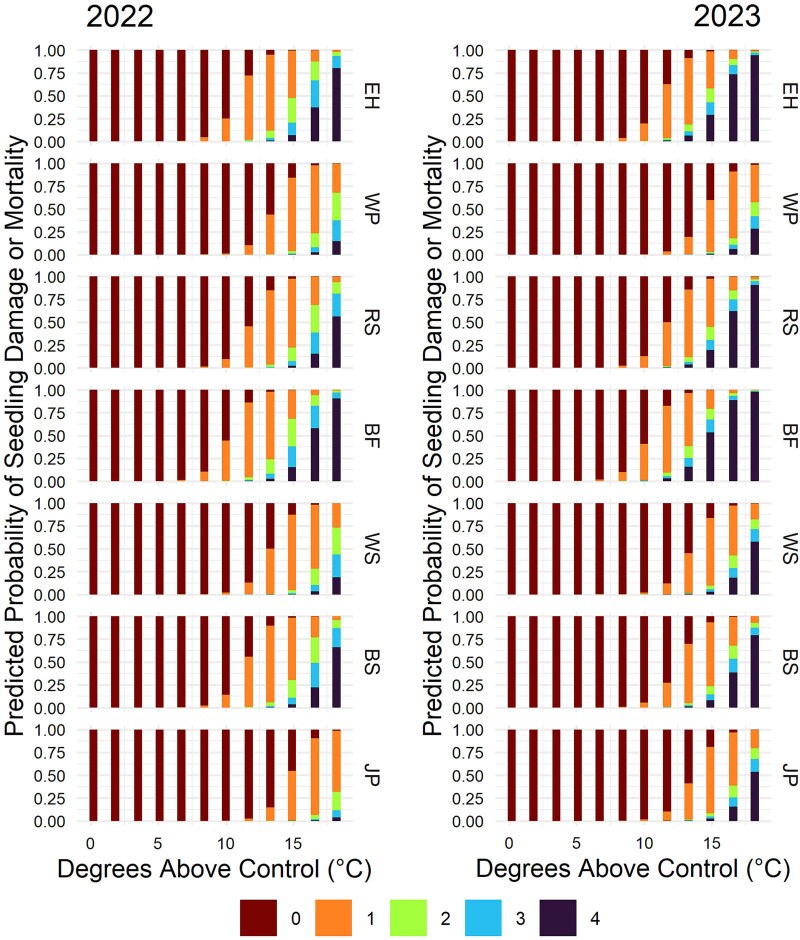
Model-predicted probability of each damage score (0: no damage; 1: <⅓ damage; 2: ≥⅓ and <⅔ damage; 3: ≥⅔ damage; 4—dead) for seedlings of balsam fir (BF), black spruce (BS), eastern hemlock (EH), jack pine (JP), red spruce (RS), white pine (WP) and white spruce (WS) under 12 levels of warming over two growing seasons. Species are ordered by ascending southern range limit latitude.

All species experienced some level of heat-induced foliar damage with increasing temperature; however, the temperature at which foliar damage became evident varied across species (Figure 2, Table 4, Table S1.2 available as Supplementary Data at *Tree Physiology* Online). The ordinal logistic regression models for the 2022 and 2023 growing seasons performed well, yielding McFadden’s pseudo-*R*^2^ values of 0.62 and 0.64, respectively. The model-predicted probabilities for the first growing season indicated that balsam fir, black spruce and eastern hemlock seedlings were likely to experience up to 33% foliar damage beginning at 11.5 °C above the control, while red spruce and white spruce were not expected to show damage until 13.1 °C above the control, and jack pine and white pine seedlings were not likely to exhibit damage until 14.7 °C above the control. After the second growing season, balsam fir and eastern hemlock exhibited the most damage in the warmer treatments, followed by red spruce, black spruce, white spruce, jack pine and white pine (Figure 2, Table 4, Table S1.2 available as Supplementary Data at *Tree Physiology* Online).

**Table 4:** Pairwise comparisons among species’ log-odds of exhibiting foliar damage with associated estimates, standard errors, *z*-ratios and *P*-values for each growing season. Each comparison is made by estimating the numerical difference between the log-odds of the two species being compared. Species are ordered by ascending southern range limit latitude. Positive estimates indicate the first species has higher cumulative log-odds, which implies a greater likelihood of being in higher damage categories. Statistically significant differences are indicated in bold. BF, balsam fir; BS, black spruce; EH, eastern hemlock; JP, jack pine; RS, red spruce; WP, white pine; WS white spruce.

Comparison	First growing season				Second growing season			
	Estimate	SE	*Z*-ratio	*P*-value	Estimate	SE	*Z*-ratio	*P*-value
EH/WP	**3.122**	**0.446**	**6.998**	**<0.001**	**3.75**	**0.665**	**5.639**	**<0.001**
EH/RS	**1.146**	**0.384**	**2.984**	**0.045**	0.529	0.559	0.946	0.965
EH/BF	−0.855	0.378	−2.261	0.263	−1.018	0.551	−1.846	0.517
EH/WS	**2.875**	**0.43**	**6.689**	**<0.001**	**2.527**	**0.624**	**4.049**	**0.001**
EH/BS	0.734	0.379	1.94	0.454	1.499	0.617	2.429	0.187
EH/JP	**4.631**	**0.512**	**9.051**	**<0.001**	**2.694**	**0.639**	**4.218**	**0.001**
WP/RS	**−1.976**	**0.438**	**−4.509**	**<0.001**	**−3.221**	**0.649**	**−4.965**	**<0.001**
WP/BF	**−3.977**	**0.469**	**−8.481**	**<0.001**	**−4.768**	**0.709**	**−6.728**	**<0.001**
WP/WS	−0.247	0.441	−0.561	0.998	−1.223	0.612	−2	0.415
WP/BS	**−2.388**	**0.439**	**−5.438**	**<0.001**	**−2.251**	**0.663**	**−3.394**	**0.012**
WP/JP	**1.509**	**0.481**	**3.136**	**0.029**	−1.056	0.63	−1.676	0.632
RS/BF	**−2.001**	**0.398**	**−5.025**	**<0.001**	−1.547	0.564	−2.743	0.088
RS/WS	**1.729**	**0.424**	**4.077**	**<0.001**	**1.998**	**0.613**	**3.258**	**0.019**
RS/BS	−0.412	0.387	−1.064	0.938	0.97	0.614	1.579	0.696
RS/JP	**3.485**	**0.496**	**7.02**	**<0.001**	**2.165**	**0.629**	**3.445**	**0.010**
BF/WS	**3.729**	**0.452**	**8.254**	**<0.001**	**3.545**	**0.658**	**5.383**	**<0.001**
BF/BS	**1.589**	**0.391**	**4.063**	**0.001**	**2.516**	**0.632**	**3.981**	**0.001**
BF/JP	**5.486**	**0.539**	**10.182**	**<0.001**	**3.712**	**0.67**	**5.54**	**<0.001**
WS/BS	**−2.14**	**0.424**	**−5.047**	**<0.001**	−1.028	0.64	−1.608	0.677
WS/JP	**1.756**	**0.483**	**3.638**	**0.005**	0.167	0.62	0.27	1.00
BS/JP	**3.897**	**0.5**	**7.8**	**<0.001**	1.196	0.652	1.833	0.525

During the first growing season, balsam fir, black spruce, eastern hemlock and red spruce in the hottest treatment experienced 100%, 100%, 80% and 90% mortality, respectively, while white spruce experienced 40%, and jack pine and white pine exhibited no mortality (Figure S1.2 available as Supplementary Data at *Tree Physiology* Online). Although the jack pine in the hottest treatment appeared to tolerate high temperatures during the first growing season (Figure 2), 5 of the 6 seedlings that remained (4 of the original 10 were removed for biomass measurements) died over the winter between growing seasons (Figure S1.2 available as Supplementary Data at *Tree Physiology* Online). During the second growing season, two of the four remaining white spruce seedlings in the hottest treatment died, while the remaining jack pine and white pine seedlings survived. The majority of the remaining balsam fir, eastern hemlock and red spruce seedlings in the top three hottest treatments died during the second growing season, which aligns with the level of heat sensitivity they exhibited.

**Figure 3:**
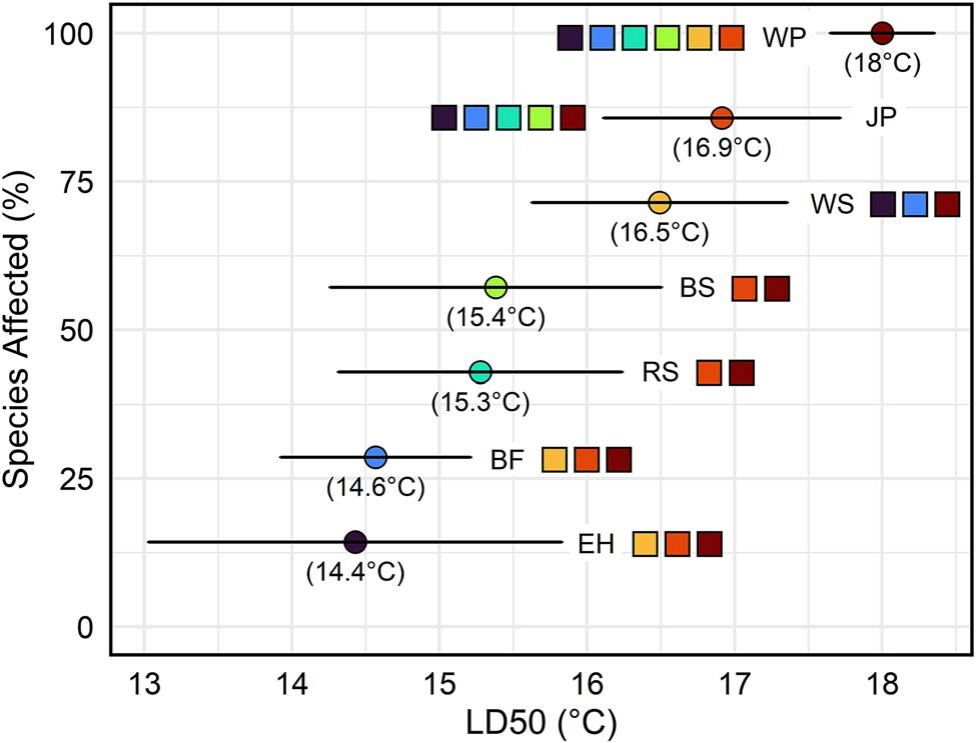
Species sensitivity distribution graph showing two-parameter log-logistic model-predicted LD50s of balsam fir (BF), black spruce (BS), eastern hemlock (EH), jack pine (JP), red spruce (RS), white pine (WP) and white spruce (WS) seedlings over the entire duration of the experiment (two growing seasons). The *y*-axis represents the cumulative percentage of the tested species that have reached 50% mortality. Temperature above control at which 50% mortality occurred (LD50) is listed in brackets under each point. The horizontal lines around each point represent the 95% confidence interval for the LD50 of each species. The colored squares next to each species show which other species differ significantly; square colors correspond to the species’ point colors.

The two-parameter log-logistic dose response model that included species as a factor provided a better fit than the null model and indicated significant differences among species’ LD50s (temperature at which 50% mortality occurs) in response to warming over two growing seasons (Figure 3, Table S1.3 available as Supplementary Data at *Tree Physiology* Online). White pine’s LD50 was 18.0 °C above the control (approximately corresponding to treatment 12, 43.0 °C maximum experienced temperature), which was significantly higher than those of all other species (*P* < 0.05). At 14.4 and 14.6 °C above the control (approximately corresponding to treatment 10, 39.7 °C maximum experienced temperature), eastern hemlock and balsam fir LD50s were the lowest, respectively. Their LD50s differed significantly from jack pine, white pine and white spruce (*P* < 0.05). Although white spruce LD50 was the highest of the spruce species, none of the spruce LD50s differed significantly from each other and were all positioned centrally within the group of species, below both pine species. Jack pine, white pine and white spruce did not reach 100% mortality in the hottest treatment, so inferences drawn from the LD50s of these species may be less accurate.

**Figure 4:**
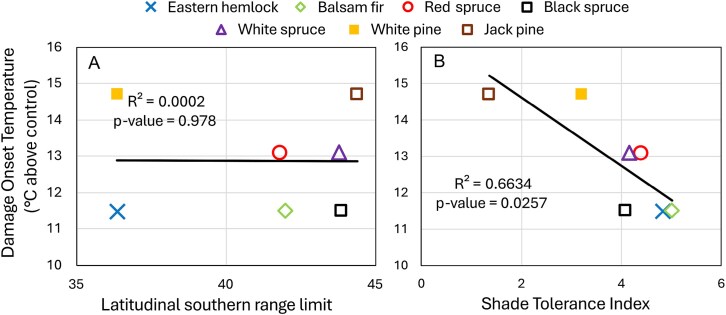
Temperature above control at which foliar damage first occurred as a function of (A) latitudinal southern range limit and (B) species shade tolerance. Shade tolerance values (0 (no tolerance) to 5 (maximal tolerance)) were taken from Niinemets and Valladares (2006), and southern range limits are based on McKenney et al. (2007).

We found a strong and statistically significant relationship between species’ shade tolerance and the temperature at which foliar heat damage first occurred (*P* < 0.05, *R*^2^ = 0.66; Figure 4), indicating that species with higher shade tolerances tended to experience damage at lower temperatures. In contrast, the latitude of the southern range limit was not significantly correlated with foliar heat damage (*P* = 0.978, *R*^2^ = 0.0002), suggesting that a species’ range limit alone is not a reliable predictor of thermal sensitivity.

### Gas exchange responses to warming treatments

**Figure 5:**
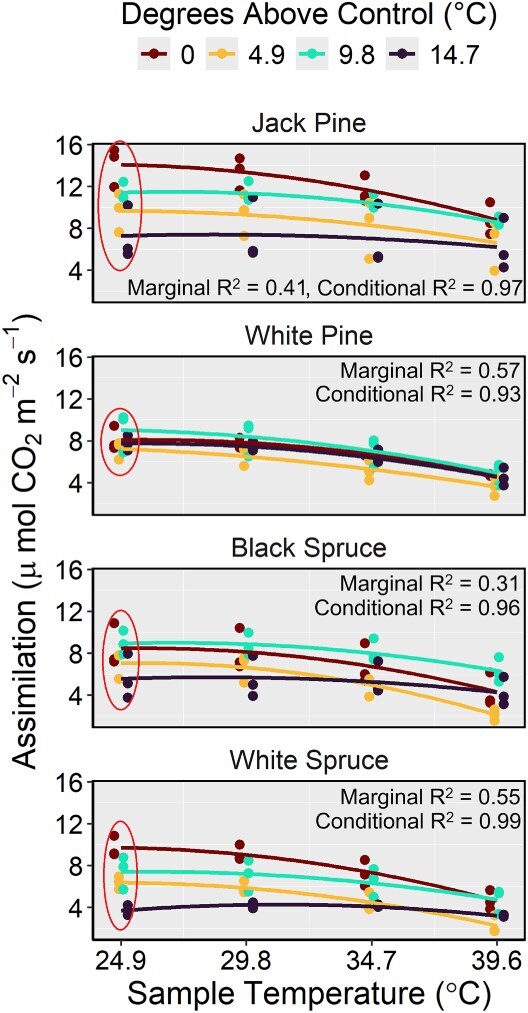
Temperature response curves for assimilation of black spruce, jack pine, white pine and white spruce seedlings. Curves were produced with species-specific models, and the associated marginal and conditional *R*^2^ values are indicated in each panel. Line colour represents the treatment from which the seedlings came (described here as degrees above control (°C)), and the sample temperature indicates the temperature inside the LI-6800 cuvette at the time of sampling. Note the large spread in jack pine, black spruce and white spruce assimilation rates from different treatments under the 24.9 °C sample temperature, relative to those of white pine (highlighted with ellipses).

Comparisons between linear and quadratic *A_net_* model forms revealed lower AICc values for the quadratic models for all species (Table S1.4 available as Supplementary Data at *Tree Physiology* Online), signifying a curvilinear response to sample temperature. The *A_net_* values of all species exhibited significant reductions over increasing sample temperature (Figure 5, Table 5), while treatment temperature did not appear to affect *A_net_* of any species as a main effect. However, we observed strong interactions between temperature treatment and sample temperature on the *A_net_* of black spruce, jack pine and white spruce (Figure 5, Table 5), indicating that their response to increasing sample temperature varied depending on the temperature treatment from which the seedlings originated. Specifically, black spruce, jack pine and white spruce seedlings from the warmest treatment exhibited lower *A_net_* values than seedlings from cooler treatments when measured under the 24.9 °C sample temperature, while little variation was observed under warmer sample temperatures. In contrast, no interactive effect between treatment temperature and sample temperature on *A_net_* of white pine seedlings was detected (Figure 5, Table 5), suggesting that the carbon assimilation capacity of white pine seedlings did not differ among treatments. The optimum temperature of white pine carbon assimilation could not be determined because *A_net_* values from all treatments consistently decreased with increasing sample temperature (Figure 5, Table S1.5 available as Supplementary Data at *Tree Physiology* Online), indicating the optimum temperature of white pine in this experiment did not exceed 24.9 °C. This pattern of declining *A_net_* with rising sample temperature was also observed in the other tested species.

**Table 5:** Results of the linear mixed-effects model analyzing the main and interactive effects of treatment temperature (*T*) and the linear and quadratic terms of sample temperature (*ST, ST^2^*) on the *A_net_* of black spruce, jack pine, white pine and white spruce. Significant interactions between *T* and the linear term of *ST* were observed for all species except white pine. Statistically significant differences are indicated in bold.

		Estimate	Standard error	DF	*T*-value	*P*-value
Jack pine						
	Intercept	6.087	16.166	33.981	3.950	0.059
	*T*	−0.224	0.619	33.981	−1.683	0.235
	***ST***	**1.348**	**0.991**	**32**	**−9.845**	**<0.001**
	*ST^2^*	−0.031	0.015	32	−2.032	0.051
	***T*:*ST***	**−0.021**	**0.038**	**32**	**6.905**	**<0.001**
	*T*:*ST^2^*	0.001	0.001	32	1.034	0.309
White pine						
	Intercept	0.245	14.387	33.131	2.992	0.096
	*T*	−0.030	0.551	33.131	0.198	0.862
	***ST***	**0.635**	**0.902**	**32**	**−3.933**	**<0.001**
	*ST^2^*	−0.014	0.014	32	−0.973	0.338
	*T*:*ST*	0.003	0.035	32	−0.040	0.969
	*T*:*ST^2^*	−0.0005	0.001	32	−0.090	0.929
Black spruce						
	Intercept	−12.280	15.220	33.940	1.888	0.200
	*T*	0.261	0.582	33.940	−0.306	0.789
	***ST***	**1.913**	**0.931**	**32**	**−9.705**	**<0.001**
	***ST*** ^***2***^	**−0.039**	**0.014**	**32**	**−2.719**	**0.011**
	***T*:*ST***	**−0.036**	**0.036**	**32**	**6.336**	**<0.001**
	*T*:*ST^2^*	0.001	0.001	32	1.439	0.160
White spruce						
	Intercept	6.496	8.560	29.080	3.206	0.085
	*T*	−0.437	0.328	29.080	−1.564	0.258
	***ST***	**1.073**	**0.499**	**32**	**−22.235**	**<0.001**
	***ST2***	**−0.028**	**0.008**	**32**	**−3.667**	**<0.001**
	***T*:*ST***	**−0.007**	**0.019**	**32**	**16.380**	**<0.001**
	*T*:*ST2*	0.0004	0.0003	32	1.503	0.143

**Figure 6:**
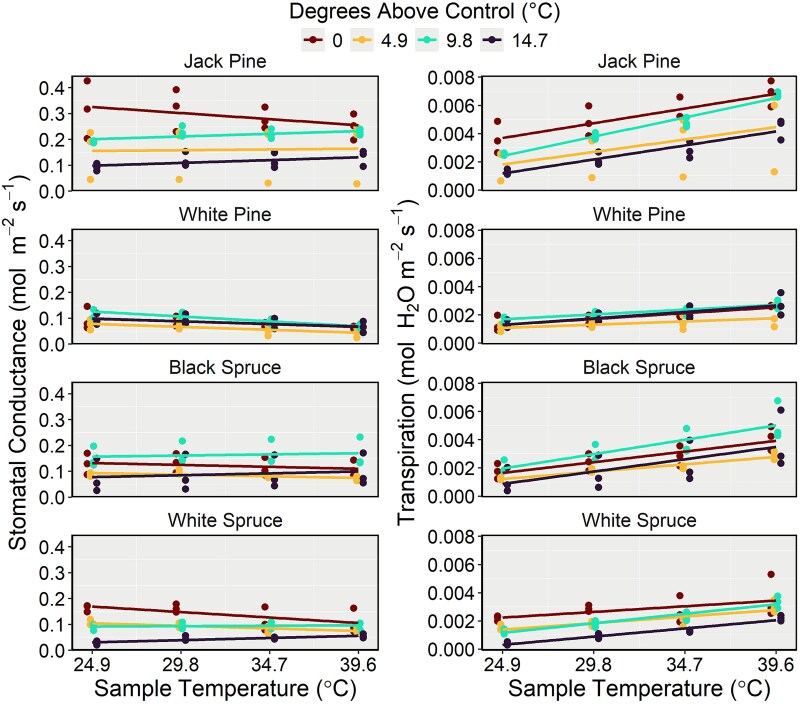
Temperature response for stomatal conductance and transpiration of black spruce, jack pine, white pine and white spruce seedlings. Line colour represents the treatment from which the seedlings came (described here as degrees above control (°C)), and the sample temperature indicates the temperature inside the LI-6800 cuvette at the time of sampling.

For the stomatal conductance and transpiration models, marginal and conditional *R*^2^ values were 0.75 and 0.95, and 0.66 and 0.95, respectively. Black spruce and white pine seedlings exhibited constant and declining *g_s_*, respectively, over the four sample temperatures, but the *g_s_* of jack pine and white spruce seedlings varied among treatments (Figure 6, Table S1.6 available as Supplementary Data at *Tree Physiology* Online). Specifically, jack pine and white spruce seedlings from the coolest treatment (treatment 1; control) reduced *g_s_* in response to warmer sample temperatures, while seedlings from the warmest treatment (treatment 10; 14.7 °C above control) increased *g_s_*. Jack pine seedlings from treatments 1 (control) and 4 (4.9 °C above control) exhibited *E* values that were significantly higher than those of black spruce, white pine and white spruce under sample temperatures 29.8, 34.7 and 39.6 °C (Figure 6, Table S1.7 available as Supplementary Data at *Tree Physiology* Online), and, although not always significant, jack pine seedlings from treatments 7 (9.8 °C above control) and 10 (14.7 °C above control) consistently exhibited the highest *E* values under every sample temperature relative to the other species.

## Discussion

After two growing seasons under 12 warming treatments, seedlings of most species exhibited enhanced above-ground growth with warming up to different temperature optima, beyond which growth declined with further warming, which agrees with trends observed in previous modeling studies (D’Orangeville et al. 2018, Wang et al. 2023). White pine peak growth occurred at higher temperatures than all other species, supporting our first hypothesis that southern species would display optimal growth under warmer temperatures. However, eastern hemlock, another southern species, exhibited either negative or negligible effects of warming on growth, while several northern species displayed varying levels of improved growth with warming above the control. This suggests that a species’ southern range limit may not consistently predict the direction of response to increasing temperature.

Although white pine exhibited the highest optimal growth temperature, our gas exchange results do not indicate that its carbon assimilation rates are higher at warmer temperatures compared with northern species (black spruce, jack pine and white spruce; Figure 5). However, treatment temperature did not influence white pine carbon assimilation, either as a main effect or through an interaction, suggesting that this species can maintain optimal rates of photosynthesis following a heatwave event, thereby maintaining its ability to grow, compete for resources and store energy. Supporting our second hypothesis, jack pine seedlings demonstrated significantly higher transpiration rates relative to the other species and the least heat-induced foliar damage during the first growing season. Heat-induced damage observed during the first growing season in our experiment illustrated what may arise during or after future heatwave events, as seedlings had not previously been exposed to high temperatures.

### Temperature effects on carbon assimilation and foliar heat stress

Declining Rubisco activity and PSII efficiency, along with increased photorespiration, dark respiration and photoinhibition, collectively contribute to a reduction in net carbon assimilation under high temperatures (Haldimann and Feller 2004, Mathur et al. 2014, Teskey et al. 2015, Perdomo et al. 2017, Guha et al. 2018, Dusenge et al. 2021, Reich et al. 2022). The effect of warming on these processes varies among species (Guha et al. 2018, Dusenge et al. 2019, 2021), but without having measured dark respiration or chlorophyll fluorescence during the period of greatest temperature stress, it is not possible to estimate how temperature affected these processes in the species we selected. While photoinhibition often occurs under high temperatures during the day, PSII efficiency in some species recovers overnight to normal levels, indicating full relaxation of non-photochemical quenching and an absence of chronic damage to PSII complexes (Guha et al. 2018). Thus, the reduction in photosynthetic capacity observed in black spruce, jack pine and white spruce seedlings

from the warmest treatment could be attributed to either transient or chronic PSII damage sustained during peak daytime temperatures. In cases where stomatal conductance declined with increasing sample temperature, stomatal limitation may have further contributed to the reduction in photosynthetic rates (Dusenge et al. 2021, Reich et al. 2022), in addition to reductions caused by declining Rubisco activity and chlorophyll degradation. However, jack pine and white spruce seedlings from the warmest treatments increased *g_s_* in response to increasing sample temperature, yet *A_net_* continued to decline, indicating that non-stomatal limitations contributed most to the reduction in photosynthesis under high temperatures. Overall, given white pine’s greater photosynthetic thermotolerance, this species may have an advantage over more northern species in the boreal–temperate ecotone (Reich et al. 2015, Guha et al. 2018) as heatwave events become more frequent (Teskey et al. 2015, Lu et al. 2023, Yin et al. 2023).

Given that adequate soil moisture was provided, the observed foliar damage and mortality under the warmest treatments are likely the result of temperature stress. Prolonged exposure to high temperatures increases thylakoid membrane fluidity and oxidative damage to chloroplasts (Mathur et al. 2014, Wang et al. 2018), severely impairing photosynthetic function (Zhang et al. 2009, Teskey et al. 2015) and eventually leading to the breakdown of chloroplasts and foliage chlorosis (Vann et al. 1994, Wang et al. 2018). Vann et al. (1994) observed chlorosis in the foliage of mature red spruce trees exposed to temperatures between 35 and 40 °C and discovered that it was caused by the breakdown of chloroplast membranes and membranes of thylakoids within. Red spruce seedlings in our experiment started to exhibit chlorosis in treatment nine (38.1 °C maximum treatment temperature, Figure 2), which aligns with the temperature range reported by Vann et al. (1994). In addition, Colombo and Timmer (1992) observed 40% foliar damage in black spruce following exposure to 40 °C, which is also consistent with our findings.

Among the species we studied, jack pine seedlings exhibited the least chlorosis under the warmest treatments after the first growing season. Having demonstrated significantly higher transpiration rates relative to other species, heat-induced foliar damage may have been mitigated due to evaporative cooling (Kolb and Robberecht 1996, Birami et al. 2018). However, under low soil water availability, a decrease in xylem water potential leads to a reduction in transpiration as stomata close, thereby eliminating

the ameliorative effects of evaporative cooling (Birami et al. 2018). In our study, soil water availability was consistently maintained at adequate levels, so whether jack pine seedlings would avoid heat-induced foliar damage under drought is unknown. Furthermore, it must be emphasized that jack pine’s ability to tolerate heat stress does not translate into greater carbon gains under future warming; seedlings from the warmer treatments exhibited reduced capacities to assimilate carbon, suggesting jack pine populations near the southern range limit will not benefit from future warming (Pedlar and McKenney 2017).

### Disentangling species’ responses to high temperature

Climate of origin has often been used to gauge a species’ temperature response: species from warmer climates are better adapted to higher temperatures than those from cooler climates (Salvucci and Crafts-Brandner 2004, Cunningham and Read 2006, Reich et al. 2015). However, when we compared the temperatures at which foliar damage first occurred with species’ southern range limits, no clear pattern was observed. A strong relationship appeared when we considered shade tolerance: light-demanding species (jack pine and white pine) showed less foliar heat stress and higher LD50s compared with shade-tolerant species (eastern hemlock, balsam fir and red spruce; Figures 3 and 4), confirming our third hypothesis. This is consistent with the results reported by Kunert and Hajek (2022), who found that light-demanding species exhibited less thermal stress than shade-tolerant species and concluded that the addition of light-demanding trees into planting schemes may improve stand stability under heatwaves. In contrast, Reich et al. (2015) found no significant effects of warming on photosynthesis or growth across species differing in shade tolerance, whether in open or understory conditions. However, the level of warming applied in their study was minimal relative to ours, and competition among species was not controlled, which would have made it difficult to isolate the effects of temperature alone on the physiology of individual species.

Given climate change projections for forests within the boreal–temperate ecotone, forests with a substantial component of shade-tolerant species may become more vulnerable to biotic and abiotic stressors. With the increased likelihood of windthrow, fire and drought events (Dai 2013, Saad et al. 2017, Taylor et al. 2020, Yin et al. 2023), more frequent heatwaves (Teskey et al. 2015, Lu et al. 2023, Yin et al. 2023) and the introduction of invasive pests like the hemlock woolly adelgid (*Adelges tsugae*; MacQuarrie et al. 2025), the likelihood of stand-clearing disturbances has never been greater. While the forest canopy may shield seedlings of shade-tolerant species from future warming, these stand-clearing disturbances would remove this protection and expose them to much hotter and drier conditions. In the future, forests dominated by shade-tolerant species may require significant intervention to successfully regenerate and recover their successional trajectories following large-scale disturbances (Ghazoul et al. 2015).

The effects of warming on seedling growth and physiology may not fully reflect the responses of mature trees to environmental stressors due to differences in physiological and structural traits. Tree seedlings have smaller non-structural carbohydrate reserves (Hartmann et al. 2018) and limited rooting depth (McDowell et al. 2008), which make them more sensitive to climatic fluctuations relative to mature trees. While seedlings were rotated weekly within the phytotrons, small microenvironmental variations among phytotrons other than temperature (e.g., air flow and light levels) may have influenced seedling growth and physiological responses. Root morphology of tree seedlings growing in plastic pots is also different from that of seedlings growing in natural forest soils. Moreover, while the 3.8 L pot size used in this experiment may have impacted total seedling biomass (Poorter et al. 2012), the effect of this limitation was equal across all treatments within species (total biomass within treatments varied across species, as expected due to differences in species-specific growth rates). As we did not compare total or relative growth values among species (only the temperature at which peak growth occurred), this limitation would have had minimal effects on our observations. However, growth of faster-growing species, like black spruce, may have been more restricted by pot size than slower-growing species, which could have restricted their maximum growth potential. With only four sample temperatures applied during gas exchange measurements, the data we collected may not have captured the full shapes of the physiological temperature responses. Although this may have limited our ability to identify species’ photosynthetic temperature optima, most *A_net_* curves declined with increasing sample temperature, indicating that the optima occurred near our lowest sample temperature (24.9 °C) or below. Overall, our findings offer valuable insights into the vulnerability of the early life stages of trees, which are critical for forest regeneration and resilience.

To our knowledge, this study examined the greatest number of tree species under the largest number of treatment levels within such a wide temperature range. This approach produced illustrative response curves for growth, heat-induced foliar damage and mortality for juveniles of seven tree species native to the boreal–temperate ecotone, providing needed empirical data to inform forest stand simulation model parameterization and development. Our results demonstrate that warming may significantly impact the growth and mortality of boreal–temperate ecotone species, with distinct differences observed among species based on both their southern range limit and shade tolerance. While light-demanding species like jack pine and white pine exhibited less damage under warmer temperatures and higher LD50s, shade-tolerant species like balsam fir, eastern hemlock and red spruce showed greater foliar damage and mortality. These responses highlight the potential vulnerability of shade-tolerant species to heat stress, especially as climate change may increase the frequency and intensity of heatwaves. Furthermore, while jack pine may possess mechanisms to mitigate oxidative damage during heatwaves, its potential to benefit from future warming in the boreal–temperate ecotone is limited compared with white pine, which demonstrated peak growth at higher temperatures and maintained photosynthetic capacity after prolonged temperature stress. These findings underscore the importance of considering both geographic range limits and species’ autecologies when predicting the future resilience of forest ecosystems in the face of climate change.

## Supplementary Material

Supplementary_materials_tpag014

## Data Availability

The data that support the findings of this study are openly available via Open Science Framework: https://osf.io/hx8qg/?view_only=845fb254ab1e42e0b41431385c9d6a4b.
