# Supplementary material for: Geographic range is a poor predictor of high-temperature responses among conifers in the boreal–temperate ecotone varying in shade tolerance
Source: Tree Physiol. 2026 Feb 21;46(3):tpag014. doi: 10.1093/treephys/tpag014 (PMC13033156; doi:10.1093/treephys/tpag014)
Supplement: Supplementary_materials_tpag014 [file supplementary_materials_tpag014.docx]

**Appendix A. Supplementary Data**

Table S1.1. Effects of both linear (T) and quadratic (T^2^) terms of temperature on seedling height and root-collar diameter increments, and above-ground biomass for the first and second growing seasons. Statistically significant terms (p < 0.05) are in bold. If the quadratic term was found to be significant, p-values were derived from models containing orthogonal polynomial terms, while T and T^2^ coefficients were estimated using models with raw polynomial terms. Quadratic terms that were not significant were removed from the model (NA). R^2^ values indicate proportion of variance explained by the final models.

| **Explanatory Variable** | **Height growth (mm)** | | | **Diameter growth (mm)** | | | **Above-ground biomass (g)** | | |
| --- | --- | --- | --- | --- | --- | --- | --- | --- | --- |
|  | **Coef.** | **P-value** | **R^2^** | **Coef.** | **P-value** | **R^2^** | **Coef.** | **P-value** | **R^2^** |
| **First growing season** | | | | | | | | | |
| **Balsam fir** |  |  |  |  |  |  |  |  |  |
| T | **-1.29** | **0.010** | **0.06** | **0.34** | **<0.001** | **0.63** | **-0.06** | **0.030** | **0.12** |
| T^2^ | NA | NA |  | **-0.01** | **<0.001** |  | NA | NA |  |
| **Black spruce** |  |  |  |  |  |  |  |  |  |
| T | 69.57 | 0.091 | **0.21** | **1.54** | **<0.001** | **0.66** | **4.49** | **0.032** | **0.33** |
| T^2^ | **-1.32** | **<0.001** |  | **-0.03** | **<0.001** |  | **-0.09** | **<0.001** |  |
| **Eastern hemlock** |  |  |  |  |  |  |  |  |  |
| T | **22.26** | **<0.001** | **0.61** | **1.05** | **<0.001** | **0.68** | -0.11 | 0.232 | 0.04 |
| T^2^ | **-0.51** | **<0.001** |  | **-0.02** | **<0.001** |  | NA | NA |  |
| **Jack pine** |  |  |  |  |  |  |  |  |  |
| T | -7.73 | 0.125 | **0.07** | -0.02 | 0.191 | 0.01 | 0.03 | 0.109 | 0.06 |
| T^2^ | **0.15** | **0.012** |  | NA | NA |  | NA | NA |  |
| **Red spruce** |  |  |  |  |  |  |  |  |  |
| T | **75.83** | **<0.001** | **0.60** | **1.04** | **<0.001** | **0.67** | **4.40** | **0.016** | **0.54** |
| T^2^ | **-1.55** | **<0.001** |  | **-0.02** | **<0.001** |  | **-0.09** | **<0.001** |  |
| **White pine** |  |  |  |  |  |  |  |  |  |
| T | **0.54** | **0.031** | **0.04** | 0.25 | 0.157 | **0.07** | 0.94 | 0.475 | **0.35** |
| T^2^ | NA | NA |  | **-0.004** | **0.015** |  | **-0.02** | **<0.001** |  |
| **White spruce** |  |  |  |  |  |  |  |  |  |
| T | 22.77 | 0.072 | **0.09** | **0.85** | **<0.001** | **0.58** | 1.83 | 0.974 | **0.29** |
| T^2^ | **-0.40** | **0.004** |  | **-0.02** | **<0.001** |  | **-0.03** | **<0.001** |  |
| **Second growing season** | | | | | | | | | |
| **Balsam fir** |  |  |  |  |  |  |  |  |  |
| T | **16.60** | **<0.001** | **0.47** | **1.26** | **<0.001** | **0.45** | **-0.44** | **<0.001** | **0.31** |
| T^2^ | **-0.44** | **0.036** |  | **-0.03** | **<0.001** |  | NA | NA |  |
| **Black spruce** |  |  |  |  |  |  |  |  |  |
| T | **-5.49** | **<0.001** | **0.22** | -0.04 | 0.178 | 0.03 | **7.90** | **0.004** | **0.27** |
| T^2^ | NA | NA |  | NA | NA |  | **-0.17** | **0.031** |  |
| **Eastern hemlock** |  |  |  |  |  |  |  |  |  |
| T | **-17.05** | **<0.001** | **0.65** | **1.60** | **<0.001** | **0.44** | -0.28 | 0.232 | 0.04 |
| T^2^ | NA | NA |  | **-0.03** | **<0.001** |  | NA | NA |  |
| **Jack pine** |  |  |  |  |  |  |  |  |  |
| T | **19.88** | **<0.001** | **0.54** | **0.65** | **<0.001** | **0.44** | **2.79** | **0.013** | **0.21** |
| T^2^ | **-0.56** | **0.030** |  | **-0.02** | **0.012** |  | **-0.06** | **0.040** |  |
| **Red spruce** |  |  |  |  |  |  |  |  |  |
| T | **39.09** | **0.004** | **0.21** | **1.08** | **<0.001** | **0.42** | **11.39** | **<0.001** | **0.48** |
| T^2^ | **-0.83** | **0.011** |  | **-0.02** | **<0.001** |  | **-0.24** | **<0.001** |  |
| **White pine** |  |  |  |  |  |  |  |  |  |
| T | **34.87** | **<0.001** | **0.43** | **0.72** | **<0.001** | **0.41** | **4.70** | **<0.001** | **0.57** |
| T^2^ | **-0.73** | **<0.001** |  | **-0.01** | **<0.001** |  | **-0.09** | **<0.001** |  |
| **White spruce** |  |  |  |  |  |  |  |  |  |
| T | **10.33** | **<0.001** | **0.63** | **1.07** | **<0.001** | **0.40** | **4.84** | **0.005** | **0.29** |
| T^2^ | **-0.34** | **0.03** |  | **-0.02** | **<0.001** |  | **-0.10** | **0.006** |  |

Table S1.2. Coefficients from an ordinal logistic regression model assessing the effect of temperature and species on damage response, with balsam fir as the reference species. Estimates, standard errors, z-values, and p-values are reported for each species relative to balsam fir. Species estimates represent the numerical change in the log-odds of damage relative to balsam fir. Statistically significant terms are in bold.

| **First growing season** | | | | |
| --- | --- | --- | --- | --- |
| **Variable** | **Estimate** | **SE** | **Z-value** | **P-value** |
| Temperature | 1.203 | 0.077 | 15.536 | **<0.001** |
| Black spruce | -1.589 | 0.391 | -4.063 | **<0.001** |
| Eastern hemlock | -0.855 | 0.378 | -2.261 | **0.024** |
| Jack pine | -5.486 | 0.539 | -10.182 | **<0.001** |
| Red spruce | -2.001 | 0.398 | -5.025 | **<0.001** |
| White pine | -3.977 | 0.469 | -8.481 | **<0.001** |
| White spruce | -3.729 | 0.452 | -8.254 | **<0.001** |
| **Second growing season** | | | | |
| **Variable** | **Estimate** | **SE** | **Z-value** | **P-value** |
| Temperature | 1.1283 | 0.108 | 10.452 | **<0.001** |
| Black spruce | -2.5163 | 0.6321 | -3.981 | **<0.001** |
| Eastern hemlock | -1.0177 | 0.5513 | -1.846 | 0.065 |
| Jack pine | -3.7121 | 0.6701 | -5.540 | **<0.001** |
| Red spruce | -1.5467 | 0.5638 | -2.743 | **0.006** |
| White pine | -4.7677 | 0.7086 | -6.728 | **<0.001** |
| White spruce | -3.5447 | 0.6585 | -5.383 | **<0.001** |

Table S1.3. Pairwise comparisons among species’ LD50s across treatments with associated estimates, standard errors (SE), t-values, and p-values. Each comparison is made using the estimated ratio of LD50 values between two species. Species are ordered by ascending southern range limit latitude. Estimates that are greater than 1.0 indicate the first species has a lower LD50 than the second. Statistically significant differences are indicated in bold.

| Comparison | Estimate | SE | T-value | P-value |
| --- | --- | --- | --- | --- |
| **EH/WP** | **0.802** | **0.041** | **-4.897** | **<0.001** |
| EH/RS | 0.945 | 0.056 | -0.995 | 0.320 |
| EH/BF | 0.990 | 0.054 | -0.177 | 0.860 |
| **EH/WS** | **0.875** | **0.049** | **-2.539** | **0.011** |
| EH/BS | 0.938 | 0.058 | -1.065 | 0.287 |
| **EH/JP** | **0.853** | **0.047** | **-3.123** | **0.002** |
| **WP/RS** | **1.178** | **0.040** | **4.497** | **<0.001** |
| **WP/BF** | **1.236** | **0.030** | **7.730** | **<0.001** |
| **WP/WS** | **1.092** | **0.031** | **2.934** | **0.003** |
| **WP/BS** | **1.170** | **0.045** | **3.771** | **<0.001** |
| **WP/JP** | **1.064** | **0.028** | **2.307** | **0.021** |
| RS/BF | 1.049 | 0.041 | 1.182 | 0.237 |
| RS/WS | 0.926 | 0.039 | -1.902 | 0.057 |
| RS/BS | 0.993 | 0.049 | -0.140 | 0.889 |
| **RS/JP** | **0.903** | **0.036** | **-2.664** | **0.008** |
| **BF/WS** | **0.883** | **0.031** | **-3.773** | **<0.001** |
| BF/BS | 0.947 | 0.041 | -1.283 | 0.200 |
| **BF/JP** | **0.861** | **0.028** | **-4.868** | **<0.001** |
| WS/BS | 1.072 | 0.049 | 1.467 | 0.142 |
| WS/JP | 0.975 | 0.035 | -0.709 | 0.478 |
| **BS/JP** | **0.909** | **0.040** | **-2.242** | **0.025** |

Table S1.4. Net assimilation model comparisons with linear and quadratic terms for sample temperature (*ST*). Models were compared using the corrected Akaike Information Criterion (AICc), with ΔAICc representing the difference in criterion values relative to the best-fitting model. Bolded values indicate the best-fitting model.

| Model | Fixed effects of *ST* | AICc | ΔAICc |
| --- | --- | --- | --- |
| Black spruce |  |  |  |
| Linear | $ST$ | 174.4 | 34.2 |
| Quadratic | $\boldsymbol{ST+}\boldsymbol{ST}^{\boldsymbol{2}}$ | **140.2** | **0** |
| Jack pine |  |  |  |
| Linear | $ST$ | 174.4 | 25.8 |
| Quadratic | $\boldsymbol{ST+}\boldsymbol{ST}^{\boldsymbol{2}}$ | **148.6** | **0** |
| White pine |  |  |  |
| Linear | $ST$ | 153.5 | 25.9 |
| Quadratic | $\boldsymbol{ST+}\boldsymbol{ST}^{\boldsymbol{2}}$ | **127.6** | **0** |
| White spruce |  |  |  |
| Linear | $ST$ | 146.7 | 54.7 |
| Quadratic | $\boldsymbol{ST+ S}\boldsymbol{T}^{\boldsymbol{2}}$ | **92.0** | **0** |

Table S1.5. Post-hoc analysis of estimated mean net assimilation (*A_net_*) values of black spruce, jack pine, white pine, and white spruce. Multiple comparisons were made using the Sidak correction among sample temperatures and within species and treatment temperatures (displayed as “Degrees above control”).

| **Sample temperature (°C)** | **Species** | **Degrees above control (°C)** | **Estimated mean *A_net_*** | **Group** |
| --- | --- | --- | --- | --- |
| 24.9 | Black Spruce | 0 | 8.53 | c |
| 29.8 | Black Spruce | 0 | 8.08 | c |
| 34.7 | Black Spruce | 0 | 6.45 | b |
| 39.6 | Black Spruce | 0 | 3.62 | a |
| 24.9 | Black Spruce | 4.9 | 7.85 | c |
| 29.8 | Black Spruce | 4.9 | 7.58 | c |
| 34.7 | Black Spruce | 4.9 | 6.31 | b |
| 39.6 | Black Spruce | 4.9 | 4.04 | a |
| 24.9 | Black Spruce | 9.8 | 7.16 | c |
| 29.8 | Black Spruce | 9.8 | 7.07 | c |
| 34.7 | Black Spruce | 9.8 | 6.17 | b |
| 39.6 | Black Spruce | 9.8 | 4.45 | a |
| 24.9 | Black Spruce | 14.7 | 6.47 | bc |
| 29.8 | Black Spruce | 14.7 | 6.57 | c |
| 34.7 | Black Spruce | 14.7 | 6.03 | b |
| 39.6 | Black Spruce | 14.7 | 4.87 | a |
| 24.9 | Jack Pine | 0 | 13.41 | d |
| 29.8 | Jack Pine | 0 | 12.72 | c |
| 34.7 | Jack Pine | 0 | 11.06 | b |
| 39.6 | Jack Pine | 0 | 8.44 | a |
| 24.9 | Jack Pine | 4.9 | 11.55 | c |
| 29.8 | Jack Pine | 4.9 | 11.14 | c |
| 34.7 | Jack Pine | 4.9 | 9.91 | b |
| 39.6 | Jack Pine | 4.9 | 7.85 | a |
| 24.9 | Jack Pine | 9.8 | 9.68 | c |
| 29.8 | Jack Pine | 9.8 | 9.56 | c |
| 34.7 | Jack Pine | 9.8 | 8.75 | b |
| 39.6 | Jack Pine | 9.8 | 7.27 | a |
| 24.9 | Jack Pine | 14.7 | 7.82 | bc |
| 29.8 | Jack Pine | 14.7 | 7.98 | c |
| 34.7 | Jack Pine | 14.7 | 7.60 | b |
| 39.6 | Jack Pine | 14.7 | 6.68 | a |
| 24.9 | White Pine | 0 | 7.92 | c |
| 29.8 | White Pine | 0 | 7.42 | c |
| 34.7 | White Pine | 0 | 6.23 | b |
| 39.6 | White Pine | 0 | 4.35 | a |
| 24.9 | White Pine | 4.9 | 7.99 | d |
| 29.8 | White Pine | 4.9 | 7.51 | c |
| 34.7 | White Pine | 4.9 | 6.32 | b |
| 39.6 | White Pine | 4.9 | 4.42 | a |
| 24.9 | White Pine | 9.8 | 8.06 | d |
| 29.8 | White Pine | 9.8 | 7.59 | c |
| 34.7 | White Pine | 9.8 | 6.40 | b |
| 39.6 | White Pine | 9.8 | 4.49 | a |
| 24.9 | White Pine | 14.7 | 8.14 | c |
| 29.8 | White Pine | 14.7 | 7.67 | c |
| 34.7 | White Pine | 14.7 | 6.48 | b |
| 39.6 | White Pine | 14.7 | 4.56 | a |
| 24.9 | White Spruce | 0 | 9.35 | d |
| 29.8 | White Spruce | 0 | 8.53 | c |
| 34.7 | White Spruce | 0 | 6.74 | b |
| 39.6 | White Spruce | 0 | 3.97 | a |
| 24.9 | White Spruce | 4.9 | 7.65 | c |
| 29.8 | White Spruce | 4.9 | 7.23 | c |
| 34.7 | White Spruce | 4.9 | 5.95 | b |
| 39.6 | White Spruce | 4.9 | 3.80 | a |
| 24.9 | White Spruce | 9.8 | 5.95 | c |
| 29.8 | White Spruce | 9.8 | 5.94 | c |
| 34.7 | White Spruce | 9.8 | 5.16 | b |
| 39.6 | White Spruce | 9.8 | 3.62 | a |
| 24.9 | White Spruce | 14.7 | 4.25 | bc |
| 29.8 | White Spruce | 14.7 | 4.64 | c |
| 34.7 | White Spruce | 14.7 | 4.37 | b |
| 39.6 | White Spruce | 14.7 | 3.45 | a |

Table S1.6. Post-hoc analysis of estimated mean stomatal conductance (*G_s_*) values of black spruce, jack pine, white pine, and white spruce. Multiple comparisons were made using the Sidak correction were made among sample temperatures and within species and treatment temperatures (displayed as “Degrees above control”).

| **Sample Temperature (°C)** | **Species** | **Degrees above control (°C)** | **Estimated mean *G_s_*** | **Group** |
| --- | --- | --- | --- | --- |
| 24.9 | Black Spruce | 0 | 0.13 | a |
| 29.8 | Black Spruce | 0 | 0.12 | a |
| 34.7 | Black Spruce | 0 | 0.11 | a |
| 39.6 | Black Spruce | 0 | 0.10 | a |
| 24.9 | Black Spruce | 4.9 | 0.12 | a |
| 29.8 | Black Spruce | 4.9 | 0.12 | a |
| 34.7 | Black Spruce | 4.9 | 0.11 | a |
| 39.6 | Black Spruce | 4.9 | 0.11 | a |
| 24.9 | Black Spruce | 9.8 | 0.11 | a |
| 29.8 | Black Spruce | 9.8 | 0.11 | a |
| 34.7 | Black Spruce | 9.8 | 0.11 | a |
| 39.6 | Black Spruce | 9.8 | 0.12 | a |
| 24.9 | Black Spruce | 14.7 | 0.10 | a |
| 29.8 | Black Spruce | 14.7 | 0.11 | a |
| 34.7 | Black Spruce | 14.7 | 0.12 | a |
| 39.6 | Black Spruce | 14.7 | 0.12 | a |
| 24.9 | Jack Pine | 0 | 0.29 | d |
| 29.8 | Jack Pine | 0 | 0.27 | c |
| 34.7 | Jack Pine | 0 | 0.26 | b |
| 39.6 | Jack Pine | 0 | 0.24 | a |
| 24.9 | Jack Pine | 4.9 | 0.23 | a |
| 29.8 | Jack Pine | 4.9 | 0.22 | a |
| 34.7 | Jack Pine | 4.9 | 0.22 | a |
| 39.6 | Jack Pine | 4.9 | 0.21 | a |
| 24.9 | Jack Pine | 9.8 | 0.16 | a |
| 29.8 | Jack Pine | 9.8 | 0.17 | a |
| 34.7 | Jack Pine | 9.8 | 0.17 | a |
| 39.6 | Jack Pine | 9.8 | 0.18 | a |
| 24.9 | Jack Pine | 14.7 | 0.10 | a |
| 29.8 | Jack Pine | 14.7 | 0.12 | b |
| 34.7 | Jack Pine | 14.7 | 0.13 | c |
| 39.6 | Jack Pine | 14.7 | 0.15 | d |
| 24.9 | White Pine | 0 | 0.09 | d |
| 29.8 | White Pine | 0 | 0.08 | c |
| 34.7 | White Pine | 0 | 0.07 | b |
| 39.6 | White Pine | 0 | 0.06 | a |
| 24.9 | White Pine | 4.9 | 0.10 | d |
| 29.8 | White Pine | 4.9 | 0.09 | c |
| 34.7 | White Pine | 4.9 | 0.07 | b |
| 39.6 | White Pine | 4.9 | 0.06 | a |
| 24.9 | White Pine | 9.8 | 0.10 | d |
| 29.8 | White Pine | 9.8 | 0.09 | c |
| 34.7 | White Pine | 9.8 | 0.08 | b |
| 39.6 | White Pine | 9.8 | 0.06 | a |
| 24.9 | White Pine | 14.7 | 0.11 | d |
| 29.8 | White Pine | 14.7 | 0.09 | c |
| 34.7 | White Pine | 14.7 | 0.08 | b |
| 39.6 | White Pine | 14.7 | 0.07 | a |
| 24.9 | White Spruce | 0 | 0.16 | d |
| 29.8 | White Spruce | 0 | 0.14 | c |
| 34.7 | White Spruce | 0 | 0.12 | b |
| 39.6 | White Spruce | 0 | 0.10 | a |
| 24.9 | White Spruce | 4.9 | 0.12 | d |
| 29.8 | White Spruce | 4.9 | 0.11 | c |
| 34.7 | White Spruce | 4.9 | 0.10 | b |
| 39.6 | White Spruce | 4.9 | 0.09 | a |
| 24.9 | White Spruce | 9.8 | 0.08 | a |
| 29.8 | White Spruce | 9.8 | 0.08 | a |
| 34.7 | White Spruce | 9.8 | 0.08 | a |
| 39.6 | White Spruce | 9.8 | 0.08 | a |
| 24.9 | White Spruce | 14.7 | 0.03 | a |
| 29.8 | White Spruce | 14.7 | 0.04 | b |
| 34.7 | White Spruce | 14.7 | 0.05 | c |
| 39.6 | White Spruce | 14.7 | 0.06 | d |

Table S1.7. Post-hoc analysis of estimated mean transpiration (*E*) values of black spruce, jack pine, white pine, and white spruce. Multiple comparisons were made using the Sidak correction were made among species and within treatment temperatures (displayed as “Degrees above control”) and sample temperature.

| **Species** | **Degrees above control (°C)** | **Sample temperature (°C)** | **Estimated mean *E*** | **Group** |
| --- | --- | --- | --- | --- |
| Black Spruce | 0 | 24.9 | 0.0017 | a |
| Jack Pine | 0 | 24.9 | 0.0033 | b |
| White Pine | 0 | 24.9 | 0.0013 | a |
| White Spruce | 0 | 24.9 | 0.0022 | ab |
| Black Spruce | 0 | 29.8 | 0.0023 | a |
| Jack Pine | 0 | 29.8 | 0.0043 | b |
| White Pine | 0 | 29.8 | 0.0016 | a |
| White Spruce | 0 | 29.8 | 0.0026 | a |
| Black Spruce | 0 | 34.7 | 0.0030 | a |
| Jack Pine | 0 | 34.7 | 0.0054 | b |
| White Pine | 0 | 34.7 | 0.0019 | a |
| White Spruce | 0 | 34.7 | 0.0030 | a |
| Black Spruce | 0 | 39.6 | 0.0037 | a |
| Jack Pine | 0 | 39.6 | 0.0064 | b |
| White Pine | 0 | 39.6 | 0.0022 | a |
| White Spruce | 0 | 39.6 | 0.0034 | a |
| Black Spruce | 4.9 | 24.9 | 0.0015 | a |
| Jack Pine | 4.9 | 24.9 | 0.0026 | b |
| White Pine | 4.9 | 24.9 | 0.0013 | a |
| White Spruce | 4.9 | 24.9 | 0.0016 | a |
| Black Spruce | 4.9 | 29.8 | 0.0023 | a |
| Jack Pine | 4.9 | 29.8 | 0.0037 | b |
| White Pine | 4.9 | 29.8 | 0.0017 | a |
| White Spruce | 4.9 | 29.8 | 0.0021 | a |
| Black Spruce | 4.9 | 34.7 | 0.0030 | b |
| Jack Pine | 4.9 | 34.7 | 0.0047 | c |
| White Pine | 4.9 | 34.7 | 0.0020 | a |
| White Spruce | 4.9 | 34.7 | 0.0026 | ab |
| Black Spruce | 4.9 | 39.6 | 0.0038 | b |
| Jack Pine | 4.9 | 39.6 | 0.0058 | c |
| White Pine | 4.9 | 39.6 | 0.0024 | a |
| White Spruce | 4.9 | 39.6 | 0.0031 | ab |
| Black Spruce | 9.8 | 24.9 | 0.0013 | a |
| Jack Pine | 9.8 | 24.9 | 0.0019 | a |
| White Pine | 9.8 | 24.9 | 0.0014 | a |
| White Spruce | 9.8 | 24.9 | 0.0010 | a |
| Black Spruce | 9.8 | 29.8 | 0.0022 | ab |
| Jack Pine | 9.8 | 29.8 | 0.0030 | b |
| White Pine | 9.8 | 29.8 | 0.0017 | a |
| White Spruce | 9.8 | 29.8 | 0.0015 | a |
| Black Spruce | 9.8 | 34.7 | 0.0030 | a |
| Jack Pine | 9.8 | 34.7 | 0.0041 | b |
| White Pine | 9.8 | 34.7 | 0.0021 | a |
| White Spruce | 9.8 | 34.7 | 0.0021 | a |
| Black Spruce | 9.8 | 39.6 | 0.0038 | b |
| Jack Pine | 9.8 | 39.6 | 0.0052 | c |
| White Pine | 9.8 | 39.6 | 0.0025 | a |
| White Spruce | 9.8 | 39.6 | 0.0027 | a |
| Black Spruce | 14.7 | 24.9 | 0.0012 | a |
| Jack Pine | 14.7 | 24.9 | 0.0012 | a |
| White Pine | 14.7 | 24.9 | 0.0014 | a |
| White Spruce | 14.7 | 24.9 | 0.0004 | a |
| Black Spruce | 14.7 | 29.8 | 0.0021 | a |
| Jack Pine | 14.7 | 29.8 | 0.0024 | a |
| White Pine | 14.7 | 29.8 | 0.0018 | a |
| White Spruce | 14.7 | 29.8 | 0.0010 | a |
| Black Spruce | 14.7 | 34.7 | 0.0030 | ab |
| Jack Pine | 14.7 | 34.7 | 0.0035 | b |
| White Pine | 14.7 | 34.7 | 0.0022 | ab |
| White Spruce | 14.7 | 34.7 | 0.0017 | a |
| Black Spruce | 14.7 | 39.6 | 0.0039 | bc |
| Jack Pine | 14.7 | 39.6 | 0.0046 | c |
| White Pine | 14.7 | 39.6 | 0.0026 | ab |
| White Spruce | 14.7 | 39.6 | 0.0023 | a |


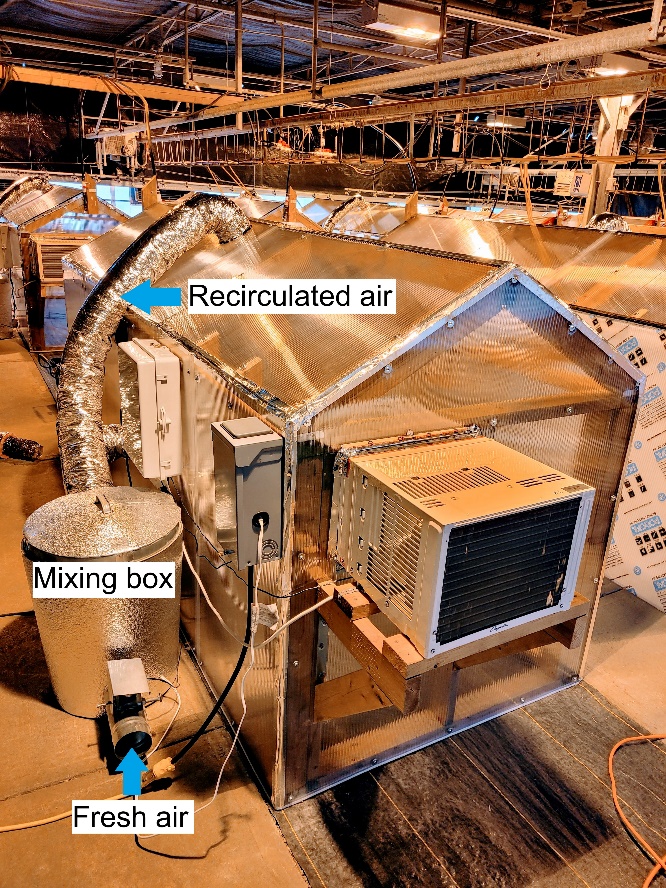

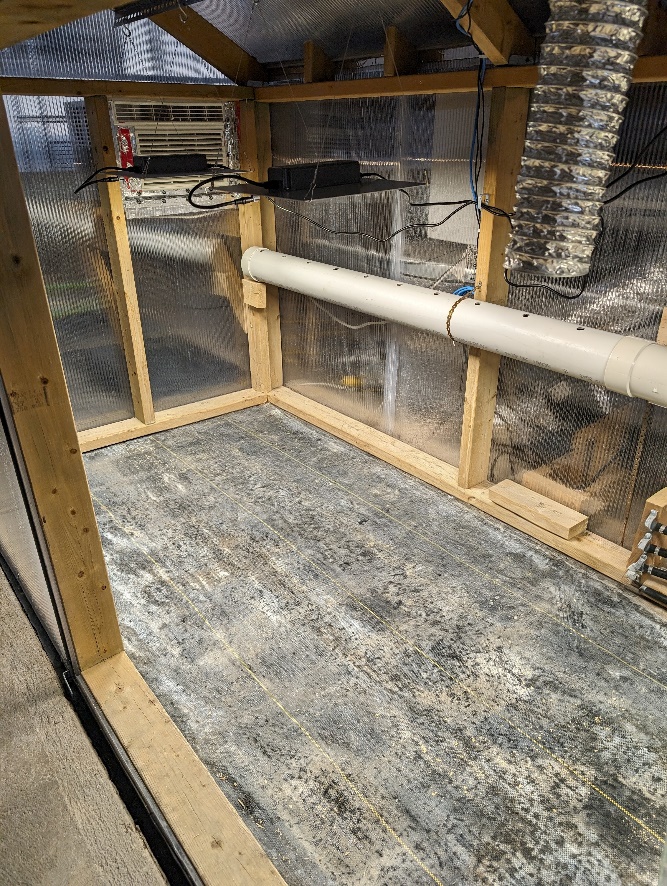


Figure S1.1. Pictures showing the outside and inside of one of the 12 phytotrons. The phytotrons are 1.0 m wide, 3.0 m long, and 1.2 m high. The ventilation system for each phytotron consists of an inline fan that draws air from a galvanized steel container (mixing box) with two inlets (fresh air and recirculated air) and one outlet. The outlet is connected to a white, perforated PVC pipe installed on the inside of the phytotron. Each phytotron has a space heater for warming and a window air conditioner for cooling. The space heater is installed inside the mixing box and the air conditioner is installed on the side of the phytotron.
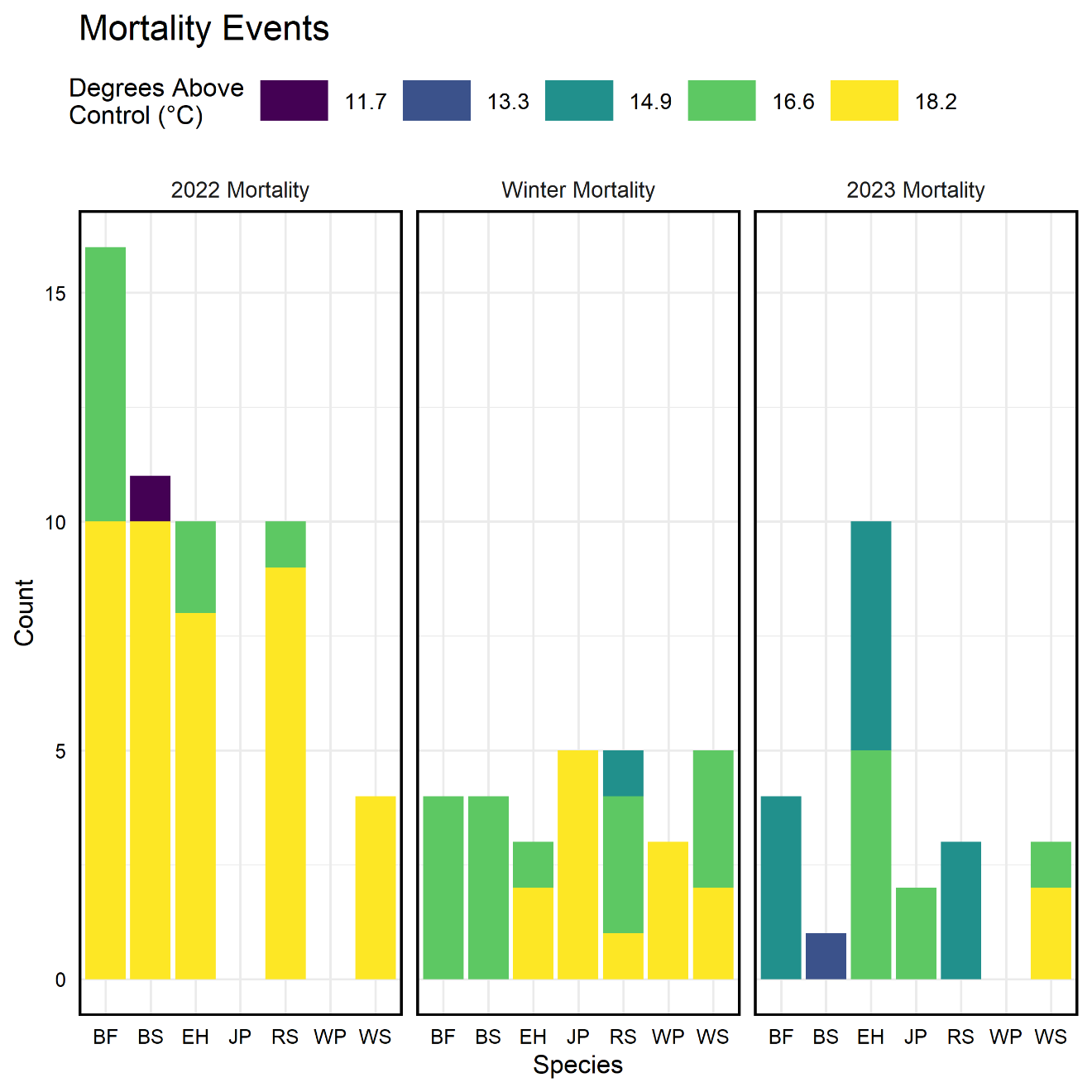


Figure S1.2. Seedling mortality events during the 2022 growing season, winter of 22/23 and the 2023 growing season of balsam fir (BF), black spruce (BS), eastern hemlock (EH), jack pine (JP), red spruce (RS), white pine (WP), and white spruce (WS) seedlings. Only treatments where mortality occurred are displayed.
